# Leptin ameliorates Aβ1-42-induced Alzheimer’s disease by suppressing inflammation via activating p-Akt signaling pathway

**DOI:** 10.1515/tnsci-2022-0270

**Published:** 2023-04-06

**Authors:** Lin Lu, Zijuan Fu, Bing Wu, Dongsen Zhang, Ying Wang

**Affiliations:** Neurology Department, Hebei Tangshan Gongren Hospital, Thangshan, 063000, China; Blood Transfusion Department, Hebei Tangshan Gongren Hospital, Tangshan, 063000, China; Emergency Department, Hebei Tangshan Gongren Hospital, Thangshan, 063000, China

**Keywords:** Alzheimer’s disease, leptin, neuronal loss, inflammation, p-Akt signaling pathway

## Abstract

**Background:**

Alzheimer’s disease (AD) is characterized by progressive neuronal loss, cognitive disorder, and memory decline. Leptin has been reported to have a neuroprotective effect on neurodegenerative diseases.

**Objective:**

Our aim was to investigate whether intraperitoneal injection of leptin has a neuroprotective effect and to explore its underlying mechanisms in the AD mouse model.

**Methods:**

Aβ1-42 was injected into male C57BL/6J mice to construct an AD mouse model, and leptin was injected intraperitoneally to cure AD. The Morris water maze test was used to investigate spatial learning ability. Neuronal loss was tested by tyrosine hydroxylase expression in the hippocampus, and terminal deoxynucleotidyl transferase mediated dUTP nick-end labeling assay was applied to detect neuronal apoptosis. Pro-inflammatory cytokine levels were monitored by RT-PCR and western blotting was selected to explore which signaling pathway leptin acted on.

**Results:**

Leptin ameliorated spatial learning impairment, restored neuronal loss and apoptosis, and inhibited pro-inflammatory cytokine expression by activating the p-Akt signaling pathway in Aβ1-42-induced AD mice.

**Conclusion:**

Leptin ameliorates Aβ1-42-induced AD by suppressing inflammation via activating the p-Akt signaling pathway.

## Graphical abstract

**Figure:**
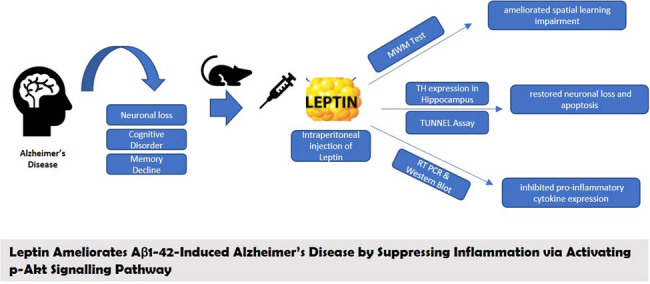

## 1 Introduction

As a common chronic neurodegenerative disease, Alzheimer’s disease (AD) is the most frequent cause of dementia characterized by progressive neuronal loss, cognitive disorder, and memory decline [1]. It affects almost 40 million patients all over the world and is regarded as one of the global health concerns of the twenty-first century [2,3]. Its main pathological features are the formation of amyloid plaques resulting from extracellular β-amyloid (Aβ) deposition and intracellular neurofibrillary tangles due to hyperphosphorylated tau in neurons [4,5]. The diagnosis of AD is still mainly based on the clinical presentation examined by the doctor; however, thanks to the advances of several new criteria, for example, imaging and fluid biomarkers can aid diagnosis as well. Although researchers are attempting to relieve the pathological changes within the brain, current treatment is primarily targeted toward symptomatic therapy [6,7] such as memantine which is available for mild to moderate AD. However, a treatment capable to stop or modify the course of AD is still under extensive research [8]. In a similar study by Assaf et al., the efficacy of fenofibrate and pioglitazone in amyloidogenesis-induced mouse models when injected intracerebroventricularly, contributed with the combinations to induce behavioral, neurochemical, and histopathological changes in amyloidogenesis model [9]. Therapeutic prospects associated with Akt pathway is already documented [10].

Leptin, a 16 kD endocrine hormone, regulates food intake, energy metabolism, neural activity, insulin sensitivity, immunity, reproduction, and bone formation [11–13]. White adipose tissue is the main source of leptin, though it can also be produced in several other peripheral tissues, such as mammary epithelium, placenta, and skeletal muscle [14]. It has been recently reported that leptin has neuroprotective effects on ischemia [15], epilepsy [16], Parkinson’s disease [17], and even AD [18]. For instance, Tong et al. found that the consecutive injection of leptin into the lateral ventricle reversed the learning and memory deficits in the AD rat model [19].

Based on the fact that leptin can enter the brain by crossing the blood–brain barrier [20], the novelty of the study is to investigate whether intraperitoneal injection of leptin has a neuroprotective effect on the AD mouse model, and to further explore the underlying mechanisms of the neuroprotective effect of leptin in AD. Since Aβ accumulation is a central and initial event in the pathogenesis of AD, and neuroinflammation is another vital driver of AD. Moreover, Akt activation, symbolized by Akt phosphorylation (p-Akt), leads to glycogen synthesis kinase-3β phosphorylation, which inhibits NF-κB activation, resulting in the expression of proinflammatory genes which are decreased [21]. We herein proposed the hypothesis that leptin ameliorates Aβ1-42-induced AD by suppressing inflammation via targeting p-Akt signaling pathway.

## 2 Methods and materials

### 2.1 Animals

Male C57BL/6J mice were purchased from Beijing Vital River Laboratory Animal Technology Co., Ltd (Beijing, China). All the mice were maintained at a 12 h light/dark cycle with free access to water and food.

### 2.2 Modeling and administration

Male C57BL/6J mice were randomized into three groups: sham group, AD model group, and leptin + AD model group (*n* = 8 in each group). These mice were placed in the mouse brain stereotaxic apparatus after being anesthetized by 45 mg/kg pentobarbital sodium injected intraperitoneally. Aβ1-42 of 4 g/L was dissolved in PBS and incubated at 37°C for 36 h to cause aggregation. All the mice in both the AD model group and the leptin + AD model group were injected with 1 μL Aβ1-42 into bilateral CA1 subregion (3.8 mm posterior to bregma, 2.5 mm lateral to the midline, and 3 mm beneath the bregma) [22], and the mice in the sham group were injected with 1 μL PBS into bilateral CA1 subregion. After surgery, the mice of the leptin + AD model group were intraperitoneally injected with leptin every day for 4 weeks (1 mg/kg) [23].

### 2.3 Morris water maze (MWM) test

The MWM test was used to investigate the spatial learning ability of the mice of each group. The test basically records the learning capacity and visuospatial memory of animals. A pool containing half-filled water was maintained at room temperature with divisions consisting of four equal quadrants and two threads perpendicular were fixed to the rim of the pool. The Mean Escape Latency was measured which is the time taken by each mouse to find the hidden platform during the trials performed over 4 days. On Day 5, the mice underwent a probe-trial session with the platforms removed from the pool and every mouse was allowed to explore the pool for 60 s. In brief, each mouse performed four trials every day, in which each mouse was allowed to look for the underwater platform for a maximum of 60 s and kept there for 10 s. Once the mouse could not find the submerged platform within 60 s, it should be guided to the platform by the experimenter and stayed there for 30 s. This procedure was conducted each time 1 day for 5 consecutive days. The tracking system was used to record the escape latency (time of finding the submerged platform). Animals were sacrificed after 5 days of MWM, and the hippocampus was isolated from the brain after euthanizing. The samples were fixed in 10% formaldehyde in PBS for 6 h [24].

### 2.4 Terminal deoxynucleotidyl transferase mediated dUTP nick-end labeling (TUNEL) assay

We isolated the hippocampus from the brain after the mice were euthanized, then it was fixed in 10% formaldehyde in PBS for 6 h, and it was finally embedded in paraffin blocks. Tissue was cut into slices, which were subsequently deparaffinized and dehydrated. After permeabilization by 20 μg/mL Proteinase K solution, the tissue slices were equilibrated in equilibration buffer and then incubated with Alexa Fluor 488-12-dUTP labeling mix and terminal deoxynucleotidyl transferase. All the nuclei were stained by DAPI, followed by the visualization of apoptotic cells under fluorescence microscopy. Finally, the apoptotic rate was evaluated by Image-Pro Plus 6 software [25].

### 2.5 RT-PCR

After the hippocampus tissues were homogenized, we removed the insoluble material and isolated total RNA from the homogenized samples by TRIZOL extraction. cDNA was synthesized from 3 μg of total RNA. According to the manufacturer’s instructions for a Universal SYBR qPCR Master Mix, real-time PCR was performed using the 1 μL cDNA template. The threshold cycles (*C*_T_) of all target genes were normalized to mouse β-Actin mRNA [26]. The primers for different genes are as follows:

**Table:** 

Gene name			Gene sequence
β-Actin	Forward	5′ CCAGCCTTCCTTCTTGGGTA 3′	
	Reverse	5′ CAATGCCTGGGTACATGGTG 3′	
IL-1β	Forward	5′ GAAATGCCACCTTTTGACAGTG 3′	
	Reverse	5′ TGGATGCTCTCATCAGGACAG 3′	
IL-6	Forward	5′ GACTGATGCTGGTGACAACC 3′	
	Reverse	5′ AGACAGGTCTGTTGGGAGTG 3′	
TNF-α	Forward	5′ ATGAGCACAGAAAGCATGATCCG 3′	
	Reverse	5′ AGAGGCTGAGACATAGGCAC 3′	

### 2.6 Western blotting

Forty micrograms of protein extracted from the hippocampus was separated in a 12% polyacrylamide gel. The protein bands on the electrophoresis gel were then transferred onto a PVDF membrane, which was incubated with 5% nonfat dry milk in Tris-buffered saline for 1 h. Subsequently, for these proteins with molecular weight, membranes were incubated overnight at 4°C with different primary antibodies (1:1,000) diluted in TBST (0.05% Tween 20, 150 mM NaCl, and 50 mM Tris, pH 7.5) with 5% nonfat dry milk. After being washed with TBST three times, all membranes were incubated in secondary antibody (horseradish peroxidase-linked anti-mouse or anti-rabbit IgG) at 1:1,000 dilutions in TBST containing 5% nonfat dry milk for 1 h. After being washed with TBST three times, a chemiluminescence HRP substrate was added to the protein bands for visualization. The ImageJ software was used to analyze the densitometric quantification of each protein band [26].

### 2.7 Statistical analysis

All data from three independent experiments were presented as the mean ± standard deviation, and statistical analyses were conducted by SPSS 24.0 software. The *t*-test was selected to compare two groups, one-way ANOVA was used for comparison among three groups, and two repeated ANOVA was chosen for comparison between two groups in the MWM test. *p* < 0.05 was considered significant.


**Ethical approval:** The research related to animals’ use has been complied with all the relevant national regulations and institutional policies for the care and use of animals. All animal experimental procedures were carried out strictly according to the guide for the protection of laboratory animals and were also approved by the hospital ethics committee in animal experiments.

## 3 Results

### 3.1 Leptin ameliorated spatial learning impairment in Aβ1-42-induced AD mice

**Figure 1:**
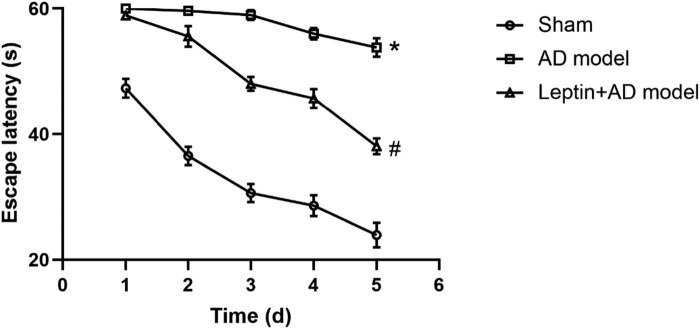
Leptin ameliorated spatial learning impairment in AD mice. ^#^ *p* < 0.05 vs AD model; ^*^ *p* < 0.05 vs sham.

The escape latencies of the sham group, AD model group, and leptin + AD model group in the MWM test were gradually decreased in consecutive trials. As shown in Figure 1, two repeated ANOVA analyses indicated that the escape latencies of the AD model group were significantly longer than those of the sham group. Furthermore, the escape latencies of the leptin + AD model group were dramatically shorter than those of the AD model group. These results above showed that leptin could reverse spatial learning deficiency.

### 3.2 Leptin restored neuronal loss and apoptosis to some extent in Aβ1-42-induced AD mice

**Figure 2:**
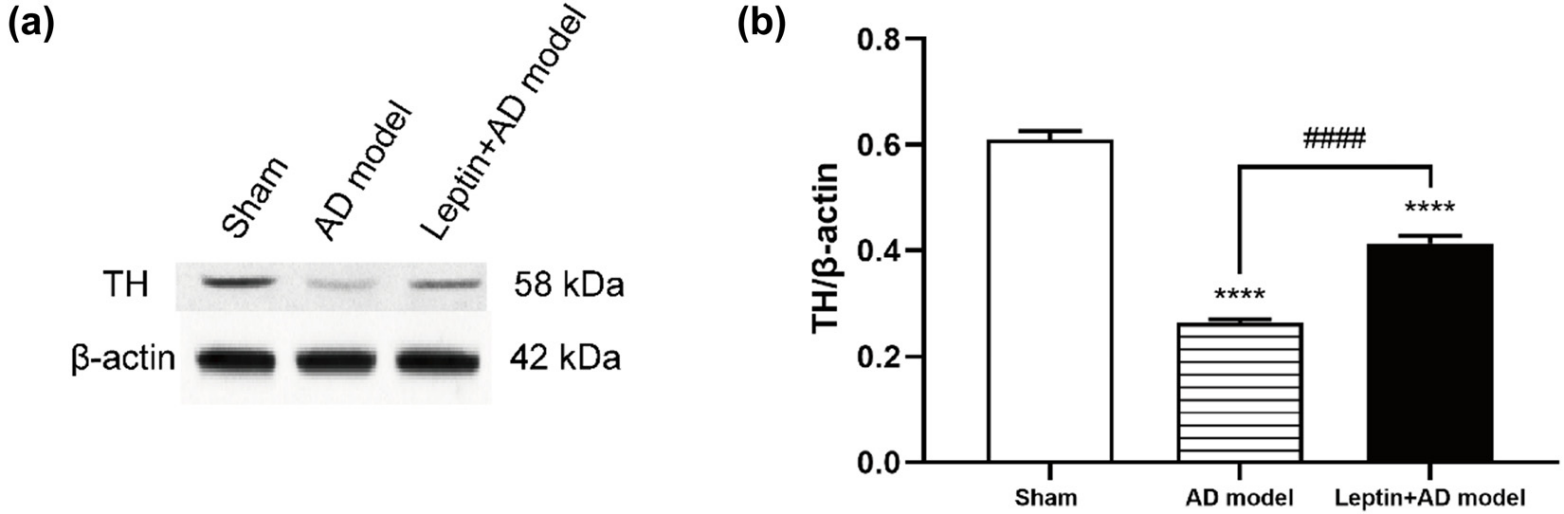
Leptin restored neuronal loss to some extent in AD mice. (a) Western blotting analysis of TH expression in the sham group, AD model group, and leptin + AD model group. (b) Semi-quantitative analysis of TH expression in the sham group, AD model group, and leptin + AD model group. ^#^ *p* < 0.05 vs AD model; ^*^ *p* < 0.05 vs sham.

To investigate the effect of leptin on the neuronal loss in the hippocampus of Aβ1-42-induced AD mice, we extracted the soluble protein from the hippocampus and applied western blotting to detect the change in the level of tyrosine hydroxylase (TH). The results in Figure 2 showed remarkably reduced expression of TH in the AD model group compared to that of the sham group; however, the leptin + AD model group significantly upregulated the TH expression compared to that of the AD model group. These results above indicated that leptin restored neuronal loss in the hippocampus to some extent caused by Aβ1-42.

**Figure 3:**
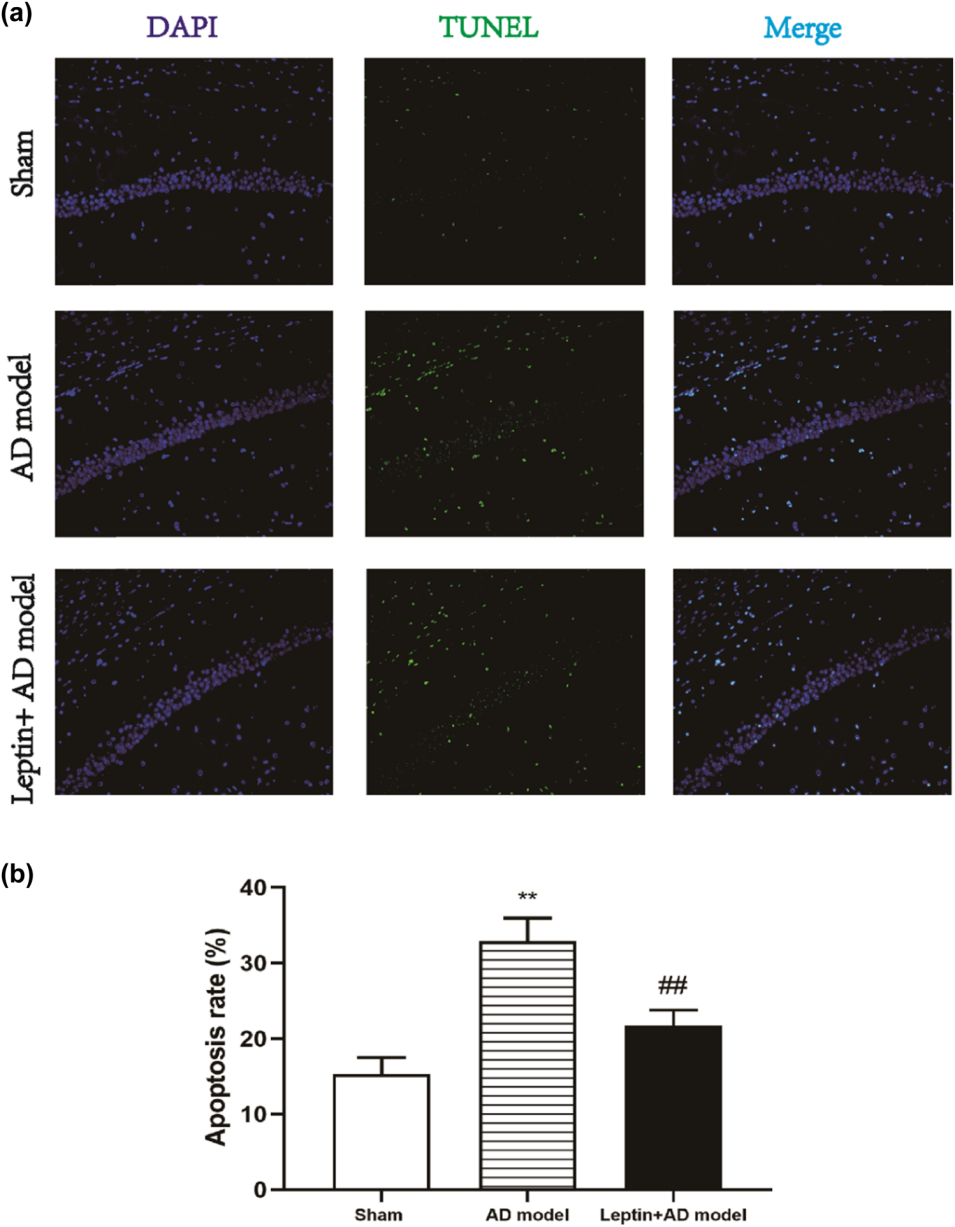
Leptin protected neuronal apoptosis to some extent in AD mice. (a) In the sham group, AD model group, and leptin + AD model group, the nuclei were stained by blue DAPI, the green TUNEL positive cells represented apoptotic cells, and merge meant co-localization of DAPI (blue) with TUNEL (green). Magnification ×200. (b) Quantitative analysis of the apoptosis rate in the sham group, AD model group, and leptin + AD model group. ^#^ *p* < 0.05 vs AD model; ^*^ *p* < 0.05 vs sham.

To ascertain whether leptin can protect the Aβ1-42-induced neuronal apoptosis in AD mice. TUNEL assay was applied to test the apoptosis rate of hippocampus tissue slices in the sham group, AD model group, and leptin + AD model group. As shown in Figure 3, the apoptosis rate was significantly increased in the AD model group when compared to the sham group, and there were also significant differences between the AD model group and the leptin + AD model group, indicating the antiapoptotic effect of leptin treatment on the Aβ1-42-induced neuronal apoptosis in AD mice.

### 3.3 Leptin inhibited pro-inflammatory cytokine expression by activating the p-Akt signaling pathway in Aβ1-42-induced AD mice

**Figure 4:**
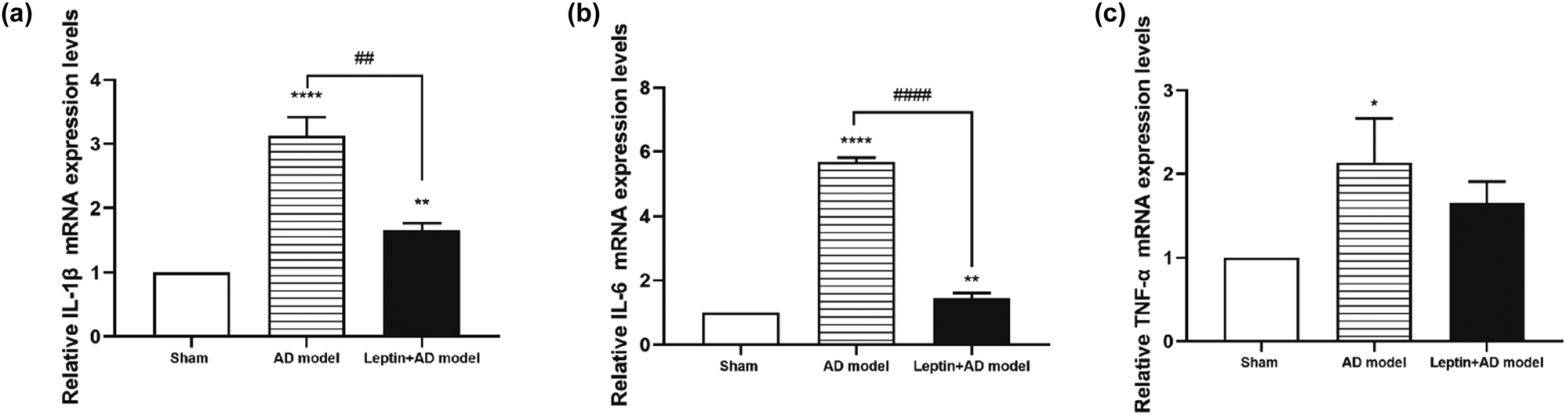
Influence of leptin on the relative mRNA expression levels of IL-1β (a), IL-6 (b), and TNF-α (c) in the sham group, AD model group, and leptin + AD model group. ^#^ *p* < 0.05 vs AD model; ^*^ *p* < 0.05 vs sham.

To evaluate the effect of leptin on pro-inflammatory cytokines, the mRNA expression of IL-1β, IL-6, and TNF-α in the hippocampus tissues was measured by real-time PCR in the sham group, AD model group, and leptin + AD model group. As shown in Figure 4, the pro-inflammatory cytokine expression was significantly increased in the AD model group compared to the sham group, and the up-regulated expression was surprisedly retarded by leptin.

**Figure 5:**
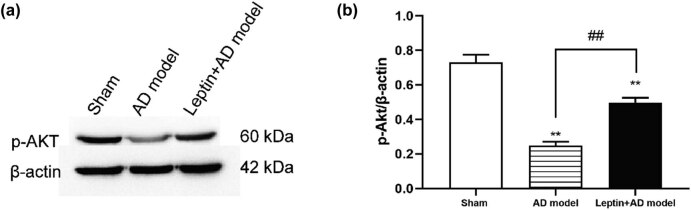
Leptin activated the p-AKT signaling pathway. (a) Western blotting analysis of p-AKT expression in the sham group, AD model group, and leptin + AD model group. (b) Semi-quantitative analysis of p-AKT expression in the sham group, AD model group, and leptin + AD model group. ^#^ *p* < 0.05 vs AD model; ^*^ *p* < 0.05 vs sham.

To explore the underlying mechanism of leptin treatment on the inflammatory effect in AD mice, the p-AKT protein expression level in the hippocampus tissues, an important inflammatory response pathway, was evaluated by western blotting in the sham group, AD model group, and leptin + AD model group. As shown in Figure 5, the p-AKT protein expression level was dramatically decreased in the AD model group compared to the sham group, but the p-AKT protein expression level in the leptin + AD model group was significantly higher than that of the AD model group. Taken together, leptin treatment decreased pro-inflammatory cytokine expression via activating the p-AKT signaling pathway.

## 4 Discussion

As the most common cause of dementia, AD is characterized by progressive amnesia, cognitive disorder, and behavioral impairment [27]. Recent research showed that leptin is associated with neurological diseases, such as neurodegenerative diseases [28]. Neuroinflammation is one of the fundamental features of AD. Deposition of Aβ deposition initiates a spectrum of microglia-activated neuroinflammation, and microglia and astrocyte activation produce the expression of various inflammatory and anti-inflammatory cytokines [29]. In one other study, the co-administration of fenofibrate and pioglitazone was shown to be effective in reducing the changes induced in the AD model mice than the administration of each intervention alone [9]. This present study was undertaken to investigate the neuroprotective effect of intraperitoneal injection of leptin on the AD mouse model. The detailed mechanisms of the neuroprotective effect of leptin in AD were also explored.

In this study, we constructed an Aβ1-42-induced AD mice model, after injection of leptin intraperitoneally, the MWM test demonstrated that leptin treatment could decrease the escape latencies, indicating that leptin treatment ameliorated spatial learning impairment in AD mice. Neuronal loss and apoptosis play a significant role in the pathological and physiological mechanisms of AD [30]. TH expression levels detection and TUNEL assay in the hippocampus confirmed that leptin treatment restored neuronal loss and apoptosis to some extent in AD mice. In addition, neuroinflammation is closely associated with the progression of AD [31]. Our results showed leptin treatment reversed the upregulated expression of pro-inflammatory cytokine (IL-1β, IL-6, and TNF-α) in AD mice. Moreover, previous research has reported that the p-Akt signaling pathway plays a vital role in inflammation [32,33]. Our results suggested that leptin treatment restored the downregulated p-Akt signaling pathway in AD mice to some extent.

However, our experimental design has some limitations. We need to use the Akt antagonist to reverse the neuroprotective effect of leptin in AD mice, to further confirm the underlying mechanisms of the neuroprotective effect of leptin in AD mice.

This study has demonstrated that leptin could ameliorate spatial learning impairment and reverses the upregulated expression of pro-inflammatory cytokine (IL-1β, IL-6, and TNF-α) via activating p-Akt signaling pathway in Aβ1-42-induced AD mice.
